# Clinical Outcome of Neonates Born to SARS-CoV-2 Positive Mothers in India: A Systematic Review and Meta-Analysis

**DOI:** 10.7759/cureus.22958

**Published:** 2022-03-08

**Authors:** Santosh K Panda, Alpana Mishra, Mona Pathak

**Affiliations:** 1 Department of Pediatrics, Kalinga Institute of Medical Sciences, Bhubaneshwar, IND; 2 Department of Community Medicine, Kalinga Institute of Medical Sciences, Bhubaneswar, IND; 3 Department of Research and Development, Kalinga Institute of Medical Sciences, Bhubaneswar, IND

**Keywords:** sars-cov-2, newborn health risk, covid 19, effect of covid 19, newborn, high risk newborn, pregnancy

## Abstract

During the SARS-CoV-2 pandemic, India accounted for 10-50% of cases reported across the world. Perinatal care from a developing country during this period has its own importance. This study was conducted to evaluate the health outcome of neonates born to SARS-CoV-2 positive mothers in India from the published literature by a systematic review and meta-analysis. Articles reporting neonates born from SARS-CoV-2 confirmed mothers in India, published in PubMed, Scopus®, and Embase® databases, were analyzed. After registration with the International Prospective Register of Systematic Reviews (PROSPERO), the study was conducted according to Preferred Reporting Items for Systematic Reviews and Meta-Analyses (PRISMA) guidelines. The primary outcomes were the mode of delivery, perinatal asphyxia, preterm birth, breastfeeding, neonatal mortality, SARS-CoV-2 infectivity among neonates of SARS-CoV-2 mothers. The pooled rate was expressed with a 95% confidence interval. Heterogeneity and study level effect size were assessed using I² statistics and DerSimonian and Laird random effect method of meta-analysis. Data analysis was made by Stata 15.1 (StataCorp LLC, College Station, Texas, USA). Total 3,551 neonates born from 3,542 SARS-CoV-2 positive mothers were included from 14 studies (four prospective and 10 retrospective studies). The pooled rates of premature birth, Caesarean delivery, breastfeeding, and neonatal mortality were 18.89%, 55.89%, 67.79%, respectively, with 12.64/1000 live births. SARS-CoV-2 positivity rate was 5.28%; 11.76% were symptomatic, and five (1.7%) died from 281 SARS-CoV-2 positive neonates. There was an increase in the number of Caesarean delivery, premature birth, and lower mortality among neonates born to SARS-CoV-2 positive mothers compared to the Indian neonatal database. Around five percent of neonates delivered to SARS-CoV-2 positive mothers were infected, and the majority of them had good clinical outcomes.

## Introduction and background

SARS-CoV-2 viral infection originated from Wuhan, China, and spread to a pandemic across the globe within a few months [1]. By June 1, 2021, 171.3 million people** **of the world were already infected with the SARS-CoV-2 virus, out of which 28.3 million (16.52%) were from India [2]. Neonates were infected during the pandemic either during the early neonatal period through possible vertical transmission or later through horizontal transmission from community or family members [3]. Neonatal health in India has always been unique as a major contributor to global live births and neonatal deaths. India is a large subcontinent, and Indian perinatal statistics are also highly heterogeneous in different states [4].

As India is one of China's neighboring countries, the upsurge of cases in India followed the peak in China and European countries. Few meta-analyses have been published regarding the health status of neonates delivered to SARS-CoV-2 positive mothers, where the majority of Indian studies were not enrolled [5-9]. In this context, this systematic review and meta-analysis intend to analyze the clinical outcomes and SARS-CoV-2 infectivity of neonates born to SARS-CoV-2 positive mothers in India from published literature.

## Review

Methods

This manuscript followed the Preferred Reporting of Systematic Review and Meta-Analysis (PRISMA) guidelines [10]. The protocol was prospectively registered in The International Prospective Register of Systematic Reviews (PROSPERO), University of York (registration number: CRD42021266632). 

All studies published from India reporting neonatal outcomes among COVID-19 positive mothers were eligible for the analysis. The outcomes under consideration were the mode of delivery, perinatal asphyxia, preterm birth, breastfeeding, neonatal mortality, SARS-CoV-2 infectivity among neonates of SARS-CoV-2 mothers.

Search strategy

A comprehensive search of PubMed, Scopus®, and Embase® databases with a pre-defined sensitive search strategy, including the search terms for SARS-CoV-2, neonatal, India, and its states, was done on June 22, 2021. The detailed search strategy is provided in the appendix. Literature published from 2020 to June 22, 2021, was included.

Study selection

All extracted records from all three databases were merged, and duplicates were removed to make a database of unique records. All extracted records were screened based on title and abstract against pre-defined inclusion criteria in the first phase independently by two authors. Articles reporting original data on neonates born to SARS-CoV-2 mothers were included without any language restriction. All records that had the eligible title and abstract screening underwent full-text review by two authors independently. Manuscripts with less than 20 neonates and lack of complete information about maternal SARS-CoV-2 confirmed status were excluded along with guidelines and systematic reviews. For any excluded article in this phase, the reason for exclusion is specified.

Data extraction

Data extraction was performed by two authors independently. Each study was critically appraised, and data was extracted from reported text/tables. The information regarding author, study site, study period, design, number of infected mothers and neonates, the timing of testing of neonates along with clinical outcomes were documented. The mode of delivery, perinatal asphyxia, preterm birth, breastfeeding, mortality, SARS-CoV-2 infectivity among neonates of SARS-CoV-2 mothers are the primary outcomes.

Statistical analysis

For all the outcomes, proportions were synthesized. Heterogeneity in study-level effect size was assessed using I² statistics [11]. Since there was moderate to high heterogeneity, the DerSimonian and Laird random effect method of the meta-analysis was used to synthesize the prevalence of binary outcomes like neonatal virus positivity, neonatal death, the study level prevalence [12]. Publication bias was visualized using a funnel plot and tested using Egger's test. All statistical analyses were conducted using Stata 15.1 (StataCorp LLC, College Station, Texas, USA).

Risk of bias

The quality of the studies was assessed using Joanna Briggs Institute (JBI) critical appraisal tool as appropriate for study, and scores were assigned (Table 1). Two authors independently assessed the quality of each of the included studies. Any discrepancy was resolved by consensus.

**Table 1:** Quality assessment of included articles using Joanna Briggs Institute (JBI) checklist

Article	Study design	Score obtained	Quality
Kumar et al. [13]	Cohort	6	Medium
Kumari et al. [14]	Case series	9	High
Anand et al. [15]	Cohort	7	Medium
Malik et al. [16]	Cohort	7	Medium
Nambiar et al. [17]	Cohort	6	Medium
Nanavati et al. [18]	Case series	10	High
Nayak et al. [19]	Case-control	9	High
Nayak et al. [20]	Cohort	6	Medium
Sehra et al. [21]	Case series	10	High
Sharma et al. [22]	Cohort	7	Medium
Singh et al. [23]	Case series	10	High
Kalamdani et al. [24]	Case series	10	High
Gupta et al. [25]	Cohort	9	High
Chakri et al. [26]	Cohort	6	Medium

Results 

**Figure 1:**
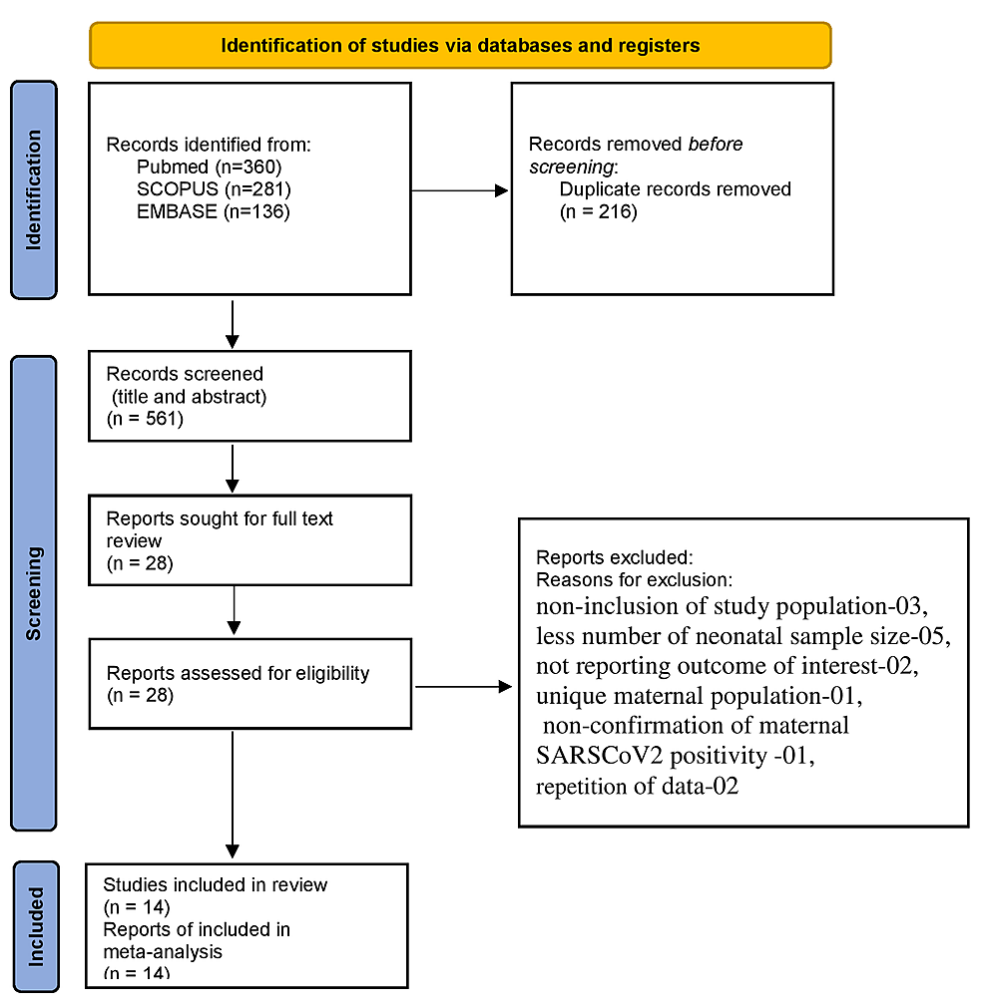
PRISMA flow chart of selected articles

A total of 561 unique records were identified after merging the extracted records from PubMed (n=360), Scopus® (n=281), and Embase® (n=136). The title and abstract of all records were screened. Out of them, 28 records were included for full-text review [13-40], and 14 records were excluded (three due to non-inclusion of the study population, five due to small neonatal sample size, two due to not reported outcomes of interest, one due to reporting on unique maternal population, one due to non-confirmation of maternal SARS-CoV-2 positivity, and two due to repetition of data). Finally, 14 Indian studies were included in this meta-analysis; a total of 3,542 SARS-CoV-2 infected pregnant women and 3,551 delivered neonates were found eligible (see Figure 1 and Table 2) [13-26].

**Table 2:** Details of the study population of the selected studies IUD - intrauterine fetal death

#	Author	Study period	Area	Study design	Time of neonatal testing	Number of SARS-CoV-2 pregnant women delivered	Total neonates	Stillbirths/ IUD	Live births
1	Nayak et al. [19]	April 1 - May 15, 2020	Mumbai	Case-control study	<24 hrs of birth	134	134	3	131
2	Nayak et al. [20]	May 1 - October 20, 2020	Bhubaneswar	Prospective study	<24 hrs of birth	162	165	2	163
3	Sharma et al. [22]	April 1 - August 31, 2020	New Delhi	Ambispective cohort	<24 hrs of birth,	41	44	0	44
					At discharge				
4	Singh et al. [23]	May 15 - November 15, 2020	Jamshedpur	Retrospective, cross-sectional	<24 hrs of birth	122	125	4	121
5	Malik et al. [16]	April 14 - July 31, 2020	Mumbai	Retrospective cross-sectional	1^st^ swab after 24 hrs of birth, 2^nd^ swab after 72 hrs	514	524	1	523
6	Kumari et al. [14]	April - August 2020	Rural Uttar Pradesh	Cross-sectional	Immediately after birth	28	29	1	28
7	Gupta et al. [25]	September 1 - November 30, 2020	Jammu - Kashmir	Retrospective cohort	<6 hrs and 48-72 hrs of birth	108	108	2	106
8	Anand et al. [15]	April 1 - July 10, 2020	New Delhi	Retrospective cohort	24 hrs of birth	68	69	4	65
										9	Sehra et al. [21]	April 13 - July 31, 2020	Jaipur	Retrospective cross-sectional	1^st^ swab after 24 hrs of birth, 2^nd^ swab after 72 hrs	120	120	nr	120
10	Nanavati et al. [24]	April 15 - July 31, 2020	Mumbai	Retrospective cross-sectional	<24 hrs of birth	122	125	0	125
11	Nambiar et al. 17]	April - November, 2020	Kerala	Retrospective cohort	Not mentioned	253	253	2	251
12	Kalamdani et al. [24]	April 1 - May 31, 2020	Mumbai	Retrospective cohort	1^st^ tested in <48 hours of birth	185	185	0	185
					if positive retested on 5^th^ day				
										13	Kumar et al. [13]	April, 2020	Multi Centric	Prospective cohort	<72 hrs of birth	1302	1330	not included	1330
14	Charki et al. [26]	May - October, 2020	North Karnataka	Prospective cohort	12-24 hrs of birth	26	28	0	0

Among 2,424 infected mothers in nine studies, 258 were symptomatic, and the pooled estimate of symptomatic mothers during delivery was 17.53% (7.35-30.71). In heterogeneity analysis, all SARS-CoV-2 positive mothers were asymptomatic in Kumari et al. [14], very few mothers were symptomatic in Kumar et al. (5.42%) [13], and Kalamdani et al. (1.62%) [24], but the majority of mothers were symptomatic in Anand et al. (73.5%) [15]. 

Only three studies had reported fetal distress in SARS-CoV-2 mothers, and a total of 50 (18.2%) out of 275 deliveries had fetal distress. It was 8/44 (18.2%), 24/106 (22.6%) and 18/125 (14.4%), respectively in Nanavati et al., Sharma et al. and Gupta et al. [18,22,25]. There were 19 (1.14%) stillbirths among 1,666 total baby births in eleven studies. Total 11 (0.42%) mothers died out of 2,596 SARS-CoV-2 infected pregnant mothers in nine studies.

**Figure 2:**
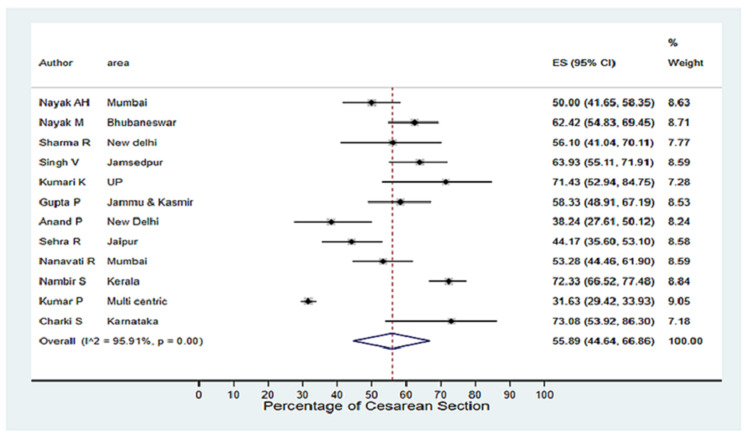
Forest plot of Caesarean delivery (pooled rates estimated considering data from 12 studies) Singh et al. [23], Kumari et al. [14], Sehra et al. [21], Nanavati et al. [18], Nayak et al. [20], Sharma et al. [22], Gupta et al. [25], Anand et al. [15], Nambiar et al. [17], Kumar et al. [13], Chakri et al. [26], Nayak et al. [19]

The Caesarean deliveries were reported in 12 out of 14 studies, and the pooled percentage of Caesarean delivery was 55.89% (44.64-66.86). As indicated in Figure 2, the heterogeneity analysis (I^2^=95.91%) shows that the Caesarean birth rate was higher in Kumari et al. (71.43 %), Nambiar et al. (72.33%), and Charki et al. (73.08%) [14,17,26] compared to majority vaginal deliveries in Kumar et al. (Caesarean deliveries 31.63%) [13] and Anand et al. (Caesarean deliveries 38.24%) [15]. A total of 103 neonates had perinatal asphyxia mentioned in 10 studies, and pooled prevalence was 4.21% (2.36-6.50). In the heterogeneity analysis (I^2^=78.75%), the rates of perinatal asphyxia were lowest in Malik et al. (1.34%) [16], Nambiar et al. (0.8%) [17], and Nayak et al. (2.29%) [19] compared to a higher rate in Kumari et al. (7.14%) [14], Nayak et al. (8.48%) [20] and Sharma et al. (11.36%) [22].

**Figure 3:**
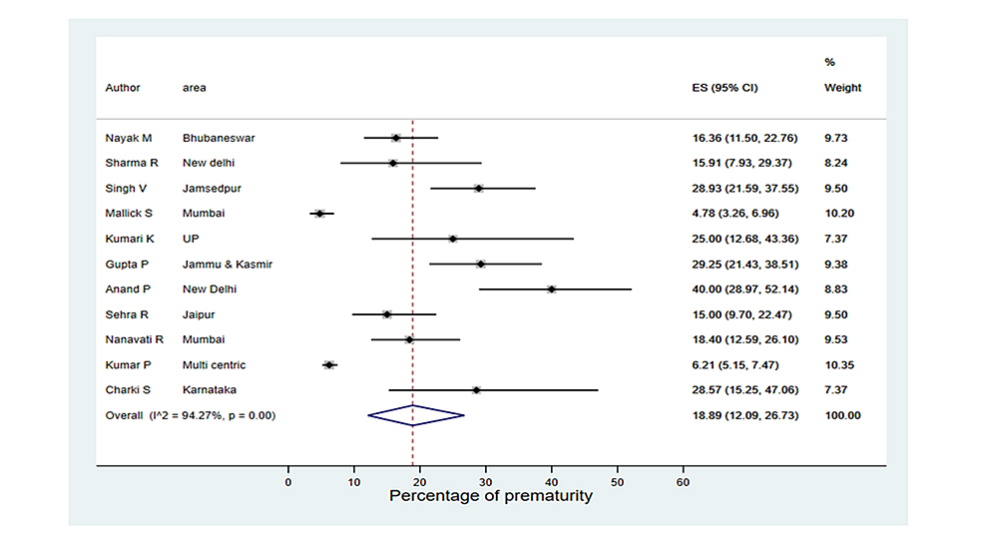
Forest plot on prematurity (estimated from 11 studies) Singh et al. [23], Kumari et al. [14], Sehra et al. [21],  Nayak et al. [20], Sharma et al. [22], Malik et al. [16], Gupta et al. [25], Anand et al. [15], Nambiar et al. [17], Kumar et al. [13], Chakri et al. [26]

As Figure 3 indicates, the pooled prevalence of prematurity was 18.89% (12.09-26.73), reported from 11 studies (310/2,984). The lower rates of preterm birth were found in Malik et al. (4.78%) and Kumar et al. (6.21%), compared to higher rates in Anand et al. (4%), Gupta et al. (29.25%), and Singh et al. (28.93%). A total of 710 neonates had low birth weight, with a pooled estimate of 28.67% (24.22-33.33). In the majority of studies, the prevalence of low birth weight (LBW) neonates was nearly 30%, whereas the prevalence of LBW in Kumar et al. and Charki et al. were 19.89% and 50%, respectively. Only two studies reported the prevalence of early gestation age as 11.3% and 23.2% in Nanavati et al. and Malik et al.. The pooled breastfeeding rate (both exclusive and mixed breastfeeding) among neonates of SARS-CoV-2 positive mothers was 67.79% (57.11%-77.64) reported in eight studies. The practice of breastfeeding rate was heterogeneous with I^2^= 93.35%; around 30% of neonates were breastfed in Sehra et al. [21] vs. 100% breastfeeding practice was executed in Kalamdani et al. [24].

**Figure 4:**
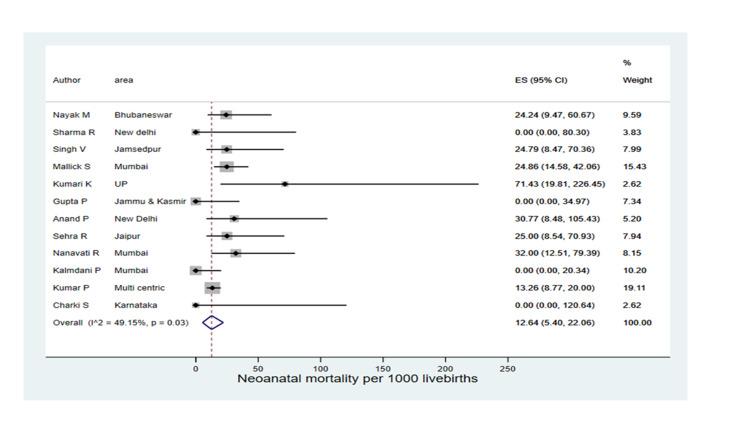
Forest plot on neonatal mortality Singh et al. [23], Kumari et al. [14], Sehra et al. [21], Nanavati et al. [18], Kalamdani et al. [24], Nayak et al. [20], Sharma et al. [22], Malik et al. [16], Gupta et al. [25], Anand et al. [15], Kumar et al. [13], Chakri et al. [26]

A total of 17.81% (11.65-24.91) live births required neonatal intensive care unit (NICU) admission in ten studies. In Nambiar et al. [17], 3.59% of neonates required NICU admission, but a higher percentage of intensive admission was found in Singh et al. (33.06%). The pooled neonatal mortality rate was 12.64 (5.40-22.06) per 1,000 live births in 12 studies, and higher neonatal mortality was reported from Kumari et al. (71.43 per 1,000 live births) as indicated in Figure 4.

**Figure 5:**
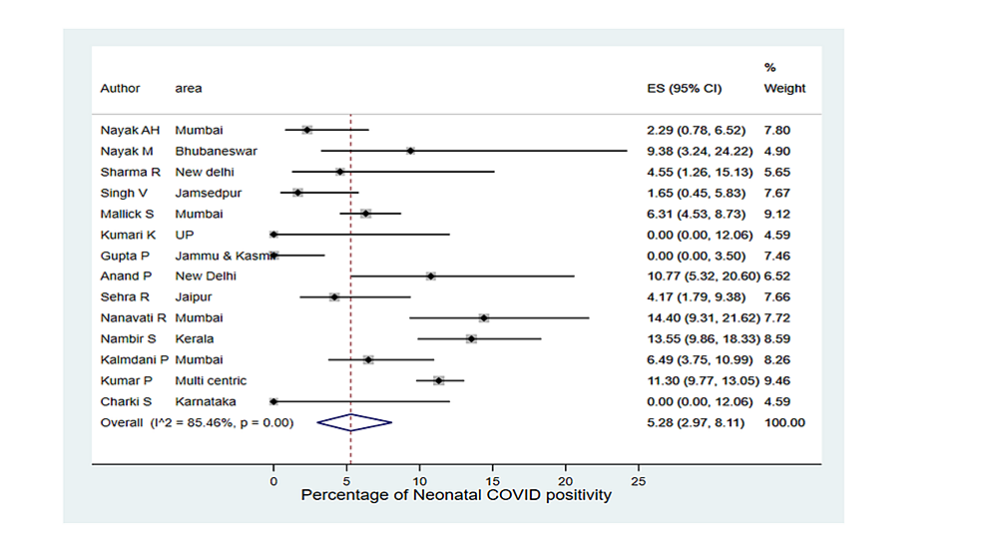
Forest plot of SARS-CoV-2 infected neonates Singh et al. [23], Kumari et al. [14], Sehra et al. [21], Nanavati et al. [18], Kalamdani et al. [24], Nayak et al. [20], Sharma et al. [22], Malik et al. [16], Gupta et al. [25], Anand et al. [15], Nambiar et al. [17], Kumar et al. [13], Chakri et al. [26], Nayak et al. [19]

A total of 281 newborns were SARS-CoV-2 infected with a pooled estimate of 5.28% infectivity (2.97-8.11) in this meta-analysis, and 242 neonates were infected within the first 72 hours of life (Figure 5). Higher neonatal infectivity was found in Anand et al., Nanavati et al., Nambiar et al., and Kumar et al., with neonatal infectivity being 10.77%, 14.40%, 13.55%, and 11.30%, respectively. There were 214 asymptomatic babies, whereas 36 required respiratory support, and out of 281 infected neonates, five died from SARS-CoV-2. Among SARS-CoV-2 neonatal cases, 11.76% (1.76-26.24) were symptomatic. In four studies, i.e., Sharma et al. (n=2), Singh et al. (n=2), Nambiar et al. (n=34), and Sehra et al.(n=5), all infected neonates were asymptomatic, whereas the rate of symptomatic cases in Malik et al., Anand et al., Nanavati et al., and Kumar et al. were 30.30%, 28.57%, 27.78%, and 21.60%, respectively. 

Apart from the primary objective of this study, there were some more clinical inputs available during the review of included manuscripts. Kumar et al. and Malik et al. compared the neonatal outcome between SARS-CoV-2 infected vs. non-infected neonates [13,16]. Shera et al., Gupta et al. compared perinatal outcomes between SARS-CoV-2 positive and negative mothers [21,24]. Anand et al., Nayak et al., Kalamdani et al., Charki et al. discussed the demographic characteristics and clinical outcomes of SARS-CoV-2 neonates [15,20,23,25]. Maternal viral loads and neonatal infectivity were studied by Anand et al. [15]. Neonatal follow-up data after hospital discharge was available in a few studies, i.e., Charki et al. followed up to two months, Sehra et al. followed up to two weeks after discharge, Anand et al. up to four weeks of life, and Kalamdani et al. followed SARS-CoV-2 infected neonates up to two months.

Discussion

This systematic review and meta-analysis is a comprehensive overview of the health status of neonates born to SARS-CoV-2 positive mothers from 14 Indian studies comprising 10 retrospective and four prospective studies [13,20,22,26]. Apart from one multicentre study [13], the rest data is from monocentric studies. Moderate to the high degree of heterogeneity was found in all primary neonatal outcome variables. There were increasing trends of Caesarean delivery and premature birth from SARS-CoV-2 positive mothers; however, the prevalence of perinatal depression and neonatal mortality was not increased in comparison to pre-COVID-19 Indian health statistics [4].

Mode of Delivery

The majority of SARS-CoV-2 mothers delivered by Caesarean section in India, similar to the high prevalence of Caesarean birth (83-85%) reported in two meta-analyses (studies mostly from China and European countries) [5,6]. In this meta-analysis, five monocentric studies reported increased Caesarean births, whereas three studies, including the multicentric study with the largest sample size, reported more vaginal birth. In Gupta et al., the Caesarean birth rate was two times higher in SARS-CoV-2 positive in comparison to SARS-CoV-2 negative mothers [25]. Elective Caesarean delivery in SARS-CoV-2 infected mothers, previous Caesarean section, presence of fetal distress, and failed induction were mentioned as indications of increased Caesarean section in some studies. Most of the study sites were the COVID-19 referral units covering a vast population, and many local logistic issues related to the pandemic such as lack of isolated space for labor, facilities for intrapartum fetal monitoring, and delayed referral could explain increasing Caesarean delivery [20,23]. Mode of delivery didn't affect neonatal infectivity in Sehra et al. [21].

Perinatal Asphyxia

The reporting of perinatal asphyxia was not uniform across the enrolled studies. The need for resuscitation, appearance, pulse, grimace, activity and respiration (APGAR) score at birth, APGAR score less than seven at five minutes, and neonates admitted for asphyxia were considered as perinatal asphyxia in the current study. The perinatal depression rate in this meta-analysis is higher compared to other systematic reviews done by Di Toro et al. and Juan et al. from overseas [5,7]. In the multicentric study, SARS-CoV-2 infected neonates required more resuscitation compared to non-infected neonates [13].

Prematurity

The preterm birth rate in India (18.8%) is slightly higher than the UK registry (16.1%) and in the USA registry (15.7%) of the American Academy of Pediatrics - The Section on Neonatal-Perinatal Medicine (AAP-SONPM) in SARS-CoV-2 mothers [8]. In two different meta-analyses by Di Toro et al. and Huntley et al. conducted during the early stage of the pandemic, the prematurity rates were 23% and 20.1%, respectively [5,41]. However, a lower prematurity burden was reported in two studies of this meta-analysis by Kumar et al. (6.2%) and Malik et al. (4.78%) [13,16]. SARS-CoV-2 infected mothers (28.3%) had more preterm babies vs. non-infected mothers (14.6%) as per the study by Gupta et al. [25]. Anand et al. reported the highest percent of premature births and also the highest percent of symptomatic mothers in this meta-analysis [15]. In a systematic review, preterm birth was the adverse outcome in SARS-CoV-2 infected pregnant mothers [9].

Breastfeeding

There is limited evidence for the existence of the SARS-CoV-2 virus in breast milk; rather, it protects neonates with SARS-CoV-2 antibodies [42]. Exclusive breastfeeding practice for neonates of SARS-CoV-2 mothers is recommended by The Federation of Obstetric and Gynecological Societies of India (FOGSI) and the National Neonatology Forum, India [43]. The breastfeeding practice was prevalent in 67.8% in India, whereas 73% of hospitals from Europe and 66.9% from the USA have practiced breastfeeding during the COVID-19 pandemic [44,45].

Around 93% of the breastfed neonates have tested negative in Anand et al. [15]. Rooming-in rather than types of feeding marginally increased the risk of infection as per the study by Kumar et al. [13]. Charki et al. reported the absence of perinatal transmission with strict adherence to respiratory and hand hygiene practice even with rooming-in and breastfeeding [26]. Lack of space for maintaining six feet distance between mother-infant dyad and compliance with appropriate hand hygiene are major concerns for possible horizontal transmission as raised by Kalamdani et al. [24]. There are favorable clinical outcomes with exclusive breastfeeding practice in all enrolled studies, including SARS-CoV-2 infected neonates as reported by Nambiar et al. and Kalamdani et al. [17,24].

Neonatal Mortality

The pooled mortality rate (12.64%) in neonates of SARS-CoV-2 mothers is lower compared to the country's projected neonatal mortality rate for 2020 [4]. The institutional delivery in a tertiary care setup and better compliance of infection control policy in COVID-19 hospitals may have helped the reduction. There is an increase in neonatal NICU admission rate among neonates of SARS-CoV-2 positive mothers compared to negative mothers, but the neonatal mortality rate is comparable between both groups in Gupta et al. [25]. Asphyxia, sepsis-like conditions and prematurity were major indications of NICU admission and neonatal mortality. There is only three early neonatal mortality per 1,000 live births in a systematic review from developed countries [8].

Neonatal SARS-CoV-2 Infectivity

In this meta-analysis, neonatal SARS-CoV-2 positivity was 5.28%, with the majority of them being infected within the first 72 hours. In contrast, two-thirds of infected neonates were diagnosed beyond 72 hours in a systematic review by Raschetti et al. [46]. Compared to the present study, there is a lower neonatal infectivity rate in the AAP registry (2.1%) of SARS-CoV-2 mothers [8].

The possibility of vertical transmission was not explored from maternal or neonatal body fluids (i.e., amniotic fluid, placenta, or IgM level in neonates, etc.) except by Sharma et al. [22]. The absence of virus in body fluids samples favors the low possibility of vertical transmission. The only early testing policy may not predict true infectivity burden when the practice of exclusive breastfeeding and rooming-in is used. The neonates of SARS-CoV-2 mothers were not infected in three studies during hospital stay [14,25,26]. In Sehra et al., the average timing of neonatal positivity beyond 72 hours may be attributed to the absence of early rooming-in and direct breastfeeding [21]. In one study, maternal viral loads didn't affect neonatal infectivity [15]. Early neonatal infectivity in this study may be influenced by neonatal care practice during testing, without any strong evidence for vertical transmission.

Neonatal SARS-CoV-2 Outcome

In this review, 11.67% of SARS-CoV-2 neonates were symptomatic, in contrast to the systemic review by Dhir et al. where they found that around 50 percent of SARS-CoV-2 infected babies were symptomatic with zero mortality [3].

It is very difficult to distinguish between early neonatal morbidity and isolated SARS-CoV-2 related symptoms, but SARS-CoV-2 infection increased neonatal morbidity in two studies [13,16]. The risk of neonatal sepsis, poor feeding, respiratory support, and deaths in SARS-CoV-2 positive neonates is increased compared to negative groups in Malik et al. [16]. In the multicentric study, SARS-CoV-2 infected neonates need more resuscitation, symptomatic and respiratory support, but there is no effect on mortality compared to those non-infected [13].

Limitation and Strength

Taking published manuscripts from different parts of India, a huge data on neonates of SARS-CoV-2 mothers were analyzed from a single country. Our study may guide the public health preparedness for perinatal outcomes in the present and future pandemic disasters in India and other low- and middle-income countries. Despite existing heterogeneity in local health arrangements and practice policy, all study sites followed the existing Indian Council of Medical Research (ICMR), National Neonatology Forum (NNF) FOGSI, COVID-19 related testing, and clinical management guidelines [47]. Case reports and case series with a sample size of less than 20 were excluded from analysis; hence reporting bias is eliminated to some extent.

This meta-analysis reported only early neonatal outcome data, mostly during the hospital stay, with few post-discharge follow-up data. As neonates of SARS-CoV-2 mothers are the targeted study participants, detailed maternal morbidity was not covered. Marked heterogeneity is reported across studies, and the appropriate statistical measures were taken to overcome that.

## Conclusions

The results of this meta-analysis based on Indian studies provides unique information for neonate SARS-CoV-2 mothers in low to middle-income countries. There is an increasing trend of Caesarean delivery and premature birth in comparison to the pre-pandemic era. The majority of SARS-CoV-2 neonates are infected within the first 72 hours and have good clinical outcomes. Due to the scarcity of strong evidence for vertical transmission, strong advocacy to abate the rising trend of Caesarean delivery should be done. As an increased need for resuscitation for neonates of SARS-CoV-2 mothers and a higher perinatal asphyxia rate were observed compared to developed countries, authors recommend aggressive fetal monitoring during the intrapartum period and timely intervention. SARS-CoV-2 infection among neonates was influenced by neonatal care practices. Breastfeeding and rooming-in practice should be encouraged with the appropriate hand and respiratory hygiene. As SARS-CoV-2 infected neonates have increased morbidity, close monitoring should be done for positive neonates. Future research should be conducted to create a population-based registry to report and compare neonatal outcomes among infected and non-infected neonates. Future research should explore the maternal SARS-CoV-2 characteristics and severity with premature delivery, fetal distress, and neonatal sickness. Further studies on predictors of symptomatic disease among SARS-CoV-2 infected neonates may be conducted in India.

## Appendices

Search strategy

PubMed

(("COVID-19"[MeSH Terms] OR "SARS-CoV-2"[MeSH Terms] OR "COVID-19"[All Fields] OR "COVID-19"[MeSH Terms] OR "covid 19 serotherapy"[All Fields] OR "covid 19 serotherapy"[Supplementary Concept] OR "covid 19 testing"[MeSH Terms] OR "SARS-CoV-2"[All Fields] OR "SARS-CoV-2"[MeSH Terms] OR "severe acute respiratory syndrome coronavirus 2"[All Fields] OR "ncov"[All Fields] OR "2019 ncov"[All Fields] OR "coronavirus"[MeSH Terms] OR "coronavirus"[All Fields] OR "cov"[All Fields]) AND (2019/11/01:3000/12/31[Date - Publication]) OR "Corona Virus disease"[All Fields] OR "coronavirus"[All Fields]) AND ("india"[MeSH Terms] OR "india"[All Fields] OR "indian"[All Fields] OR "tamil nadu"[All Fields] OR "andhra pradesh"[All Fields] OR "kerala"[All Fields] OR "goa"[All Fields] OR "karnataka"[All Fields] OR "maharashtra"[All Fields] OR "gujarat"[All Fields] OR "rajasthan"[All Fields] OR "delhi"[All Fields] OR "national capital region"[All Fields] OR "delhi ncr"[All Fields] OR "chandigarh"[All Fields] OR "punjab"[All Fields] OR "jammu"[All Fields] OR "kashmir"[All Fields] OR "himachal pradesh"[All Fields] OR "uttarakhand"[All Fields] OR "uttaranchal"[All Fields] OR "uttar pradesh"[All Fields] OR "madhya pradesh"[All Fields] OR "jharkhand"[All Fields] OR "chhattisgarh"[All Fields] OR "bihar"[All Fields] OR "orissa"[All Fields] OR "odisha"[All Fields] OR "telangana"[All Fields] OR "west bengal"[All Fields] OR "sikkim"[All Fields] OR "assam"[All Fields] OR "nagaland"[All Fields] OR "manipur"[All Fields] OR "meghalaya"[All Fields] OR "tripura"[All Fields] OR "mizoram"[All Fields] OR "arunachal pradesh"[All Fields] OR "dadra"[All Fields] OR "nagar haveli"[All Fields] OR "daman"[All Fields] OR "diu"[All Fields] OR "andaman"[All Fields] OR "nicobar"[All Fields] OR "lakshadweep"[All Fields] OR "haryana"[All Fields] OR "puducherry"[All Fields] OR "pondicherry"[All Fields] OR "andaman and nicobar"[All Fields] OR "daman and diu"[All Fields] OR "dadra and nagar haveli"[All Fields]) AND ("infant, newborn"[MeSH Major Topic] OR "neonatology"[MeSH Major Topic] OR "infant"[Text Word] OR "newborn"[Text Word] OR "neonate"[Text Word] OR "neonatal"[Text Word] OR "toddler"[Text Word] OR "baby"[Text Word] OR "babies"[Text Word] OR "paediatric"[Text Word] OR "paediatric"[Text Word] OR pregnancy)

Embase®

('coronavirus disease 2019'/exp OR 'coronavirus disease 2019') AND india:ca AND (pregnancy:ti,ab,kw OR neonatology:ti,ab,kw OR infant:ti,ab,kw OR newborn:ti,ab,kw OR neonate:ti,ab,kw OR neonatal:ti,ab,kw OR toddler:ti,ab,kw OR baby:ti,ab,kw OR babies:ti,ab,kw OR paediatric:ti,ab,kw)

Scopus®

( TITLE-ABS-KEY ( covid-19  OR  sars-cov-2  OR  ( corona  AND virus  AND disease )  OR  coronavirus )  AND  TITLE-ABS-KEY ( ( pregnancy  OR  neonatology  OR  infant  OR  newborn  OR  neonate  OR  neonatal  OR  toddler  OR  baby  OR  babies  OR  paediatric  OR  paediatric ) )  AND  TITLE-ABS-KEY ( india  OR  indian  OR  tamilnadu  OR  andhra  OR  kerala  OR  goa  OR  karnataka  OR  maharashtra  OR  gujarat  OR  rajasthan  OR  delhi  OR  chandigarh  OR  punjab  OR  jammu  OR  kashmir  OR  himachal  OR  uttarakhand  OR  uttaranchal  OR  uttar  OR  madhya  OR  jharkhand  OR  chhattisgarh  OR  bihar  OR  orissa  OR  odisha  OR  telangana  OR  "west bengal"  OR  sikkim  OR  assam  OR  nagaland  OR  manipur  OR  meghalaya  OR  tripura  OR  mizoram  OR  arunachal  OR  lakshadweep  OR  haryana  OR  puducherry  OR  pondicherry ) )
